# Polarized Raman Anisotropic Response of Collagen in Tendon: Towards 3D Orientation Mapping of Collagen in Tissues

**DOI:** 10.1371/journal.pone.0063518

**Published:** 2013-05-15

**Authors:** Leonardo Galvis, John W. C. Dunlop, Georg Duda, Peter Fratzl, Admir Masic

**Affiliations:** 1 Department of Biomaterials, Max Planck Institute of Colloids and Interfaces, Potsdam, Germany; 2 Berlin-Brandenburg School for Regenerative Therapies (BSRT), Charité Campus Virchow-Klinikum, Berlin, Germany; 3 Julius Wolff Institute & Center for Musculoskeletal Surgery, Charité - Universitätsmedizin Berlin, Berlin, Germany; University of Liverpool, United Kingdom

## Abstract

In this study, polarized Raman spectroscopy (PRS) was used to characterize the anisotropic response of the amide I band of collagen as a basis for evaluating three-dimensional collagen fibril orientation in tissues. Firstly, the response was investigated theoretically by applying classical Raman theory to collagen-like peptide crystal structures. The theoretical methodology was then tested experimentally, by measuring amide I intensity anisotropy in rat tail as a function of the orientation of the incident laser polarization. For the theoretical study, several collagen-like triple-helical peptide crystal structures obtained from the Protein Data Bank were rotated “in plane” and “out of plane” to evaluate the role of molecular orientation on the intensity of the amide I band. Collagen-like peptides exhibit a sinusoidal anisotropic response when rotated “in plane” with respect to the polarized incident laser. Maximal intensity was obtained when the polarization of the incident light is perpendicular to the molecule and minimal when parallel. In the case of “out of plane” rotation of the molecular structure a decreased anisotropic response was observed, becoming completely isotropic when the structure was perpendicular to the plane of observation. The theoretical Raman response of collagen was compared to that of alpha helical protein fragments. In contrast to collagen, alpha helices have a maximal signal when incident light is parallel to the molecule and minimal when perpendicular. For out-of-plane molecular orientations alpha-helix structures display a decreased average intensity. Results obtained from experiments on rat tail tendon are in excellent agreement with the theoretical predictions, thus demonstrating the high potential of PRS for experimental evaluation of the three-dimensional orientation of collagen fibers in biological tissues.

## Introduction

Collagen is an important structural component in many biological tissues including bone, teeth and skin [1]. It imparts toughness [2] and deformability to these tissues and by controlling its fibrillar arrangement at multiple hierarchical levels [3] organisms can produce tissues both with highly directional (anisotropic) material properties as well as more isotropic properties [4]. As such knowledge of the collagen orientation within a tissue is valuable information in order to understand and predict the tissue behaviour. Several methods can be used to measure collagen orientation, including electron microscopy [5], small angle X-ray scattering (SAXS) [6], [7], polarized light microscopy [8], and second harmonic generation microscopy [9], but they either require complex sample preparation and do not give concurrent information about the local sample chemistry. Polarized Raman spectroscopy (PRS) is a vibrational spectroscopy technique that can provide information regarding chemical composition in materials [10]. Raman spectroscopy is based on the analysis of the inelastic scattering of light interacting with molecules in which the frequency shift between the incident and the scattered light is associated with a particular vibration mode of a chemical bond. Considering their hierarchical structure from atomic up to macroscopic scale, several biological materials have already been the object of studies by PRS. For instance, PRS has been used to investigate the molecular organization of cellulose fibril in wood cells [11], the protein secondary structure organization in spider silk [12]–[14], the anisotropic response of Raman bands in collagen bundles [15], and the hydroxyapatite crystallite orientation in human enamel [16]. Furthermore, bone has also been extensively studied using PRS since several material property parameters such as mineral to organic matrix ratio can be extracted from the Raman spectrum [17]–[19].

**Figure 1:**
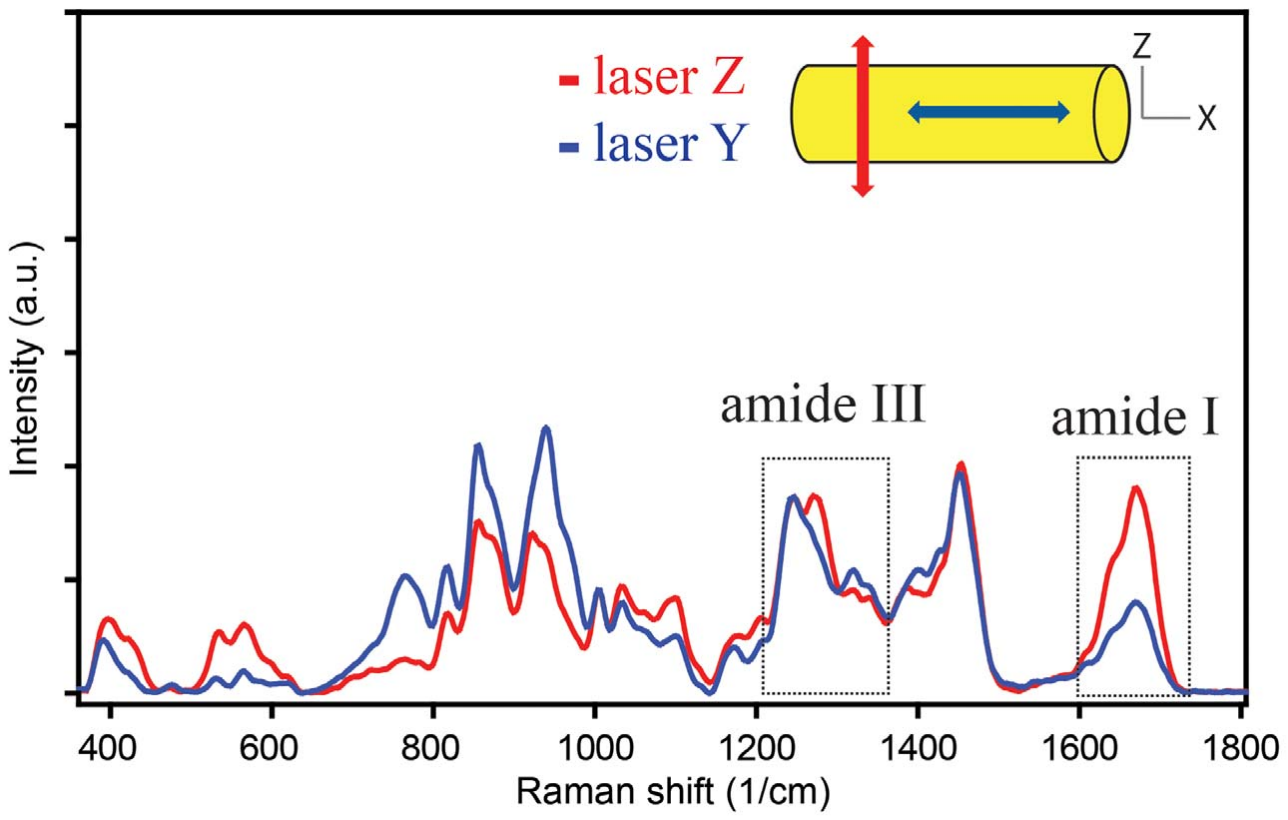
Polarized Raman spectroscopy of RTT. Spectra taken at the same spot of the sample were collected with two different laser polarization orientations [parallel (laser X, blue line) and perpendicular (laser Z, red line) to the tendon axis]. A large anisotropy of the amide I band in the two different laser to fiber configurations is due to the preferential orientation of vibrational units along the main axis of the tendon.

Confocal Raman microscopy is a non-invasive imaging technique that provides chemical information with high special resolution (0.6–1 µm) [10], [20]. The principal Raman scattering bands used to image the bone organic matrix are the amide I (∼1620–1700 cm^−1^) and amide III (1215–1300 cm^−1^) bands. The amide I band is mainly due to C = O stretching, whereas the amide III band arises from the combination of N–H bending and C–N stretching of the peptide backbone. Because the laser used to excite the sample is inherently polarized, molecular orientation effects emerging from the tensorial nature of the polarizability, a physical quantity that describes Raman scattering for a given molecular group, cannot be ignored when compositional analysis is performed [21], [22] (see examples of PRS spectra for rat tail tendon in Fig. 1). Therefore, it is important to evaluate the magnitude of such effects when highly anisotropic bands such as the amide I band are selected to perform compositional analysis, for example in bone [23]. This anisotropic response, although making data evaluation more complex, in fact enriches the information contained in Raman spectrum. This means that PRS can be potentially used to determine the molecular orientation within the sample by measuring the anisotropic Raman response of certain chemical bonds at different polarization of the incident radiation [24].

Recently, several studies have been performed to clarify orientation effects on the intensity of Raman bands associated with collagen and mineral phases in bone and other collagen based tissues [15], [21], [25]–[27]. Janko et al. [15] explored orientation dependence of collagen related bands in the Raman spectrum of rat tail tendon fibres showing strong anisotropy of several Raman lines. The anisotropy of amide I band was further exploited by Masic at al. [25] to investigate how loading induced changes of collagen orientation in the crimp regions of rat tail tendon. Kazanci. et al. [21], [23] studied orientation dependence in the intensity response of organic and mineral Raman bands of osteonal bone tissue and first indicated the use of the amide III for the determination of compositional ratios due to its smaller susceptibility to orientation effects; Gamsjaeger et al. [22] performed Raman polarization experiments in normal mouse cortical bone to determine collagen orientation as function of the animal age by using amide I band anisotropic response; more recently Falgayrac G. et al. [27] studied the fibril alignment of the lamellar bone in cortex by using PRS, taking in consideration the behaviour of specific Raman ratios.

In the present work, we studied the anisotropy of the theoretical Raman amide I band intensity within several collagen-like peptide structures along with some other helical structures, such as alpha-helices. The model was then used to extract three-dimensional (3D) orientation information of collagen molecules in native tissues as measured experimentally in rat tail tendon. The theoretical study results were in excellent agreement with the experimental data, thus showing that PRS imaging can also be used for 3D spatial orientation determination of the collagen fibrils in tissues.

## Materials and Methods

### Ethics Statement

The animal welfare as well as method of sacrifice was approved by the local authority Landesamt für Gesundheit und Soziales (Berlin, Germany).

### Rat Tail Tendon (RTT)

A fascicle of approximately 20 mm in length and 200 µm in thickness was dissected from the proximal end of the tail of a Sprague-Dawley rat aged 12 months, stretched to remove the crimp and dried at room temperature. Animals were euthanized in deep anaesthesia by intracardial injection of 1 ml Rompun.

### Raman Spectroscopy

For Raman microspectroscopy, a continuous laser beam was focused down to a micrometer sized spot on the sample through a confocal Raman microscope (CRM200, WITec, Ulm, Germany) equipped with a piezo-scanner (P-500, Physik Instrumente, Karlsruhe, Germany). A diode-pumped 785 nm near-infrared (NIR) laser excitation (Toptica Photonics AG, Graefelfing, Germany) was used in combination with a 20× (Nikon, NA = 0.4) microscope objective. The spectra were acquired using a CCD (PI-MAX, Princeton Instruments Inc., Trenton, NJ) behind a grating (300 g mm^−1^) spectrograph (Acton, Princeton Instruments Inc., Trenton, NJ) with a spectral resolution of ∼6 cm^−1^. Thirty accumulations with integration time of 1 s were used for single spot analyses. For mapping purposes the surface was scanned with steps of 2 µm integrating the signal for 0.3 s. The ScanCtrlSpectroscopyPlus (version 1.38, WITec, Ulm, Germany) and WitecProjectPlus (version 2.02, WITec, Ulm, Germany) were used for the experimental setup and spectral data processing, respectively. Chemical images were achieved by integration over defined Raman shift region in the spectrum using a sum filter. The filter calculates the intensities within the chosen borders and the background is subtracted by taking the baseline from the first to the second border. The amide I intensity was obtained by integrating the total intensity of the Amide I band (1600–1700 cm^−1^). The Raman orientation maps were produced by a non-linear least squares fitting procedure provided by Matlab 7.5 (MathWorks Inc., Natick, MA, USA) using built-in and locally written scripts [25].

### Theoretical Basis

**Figure 2:**
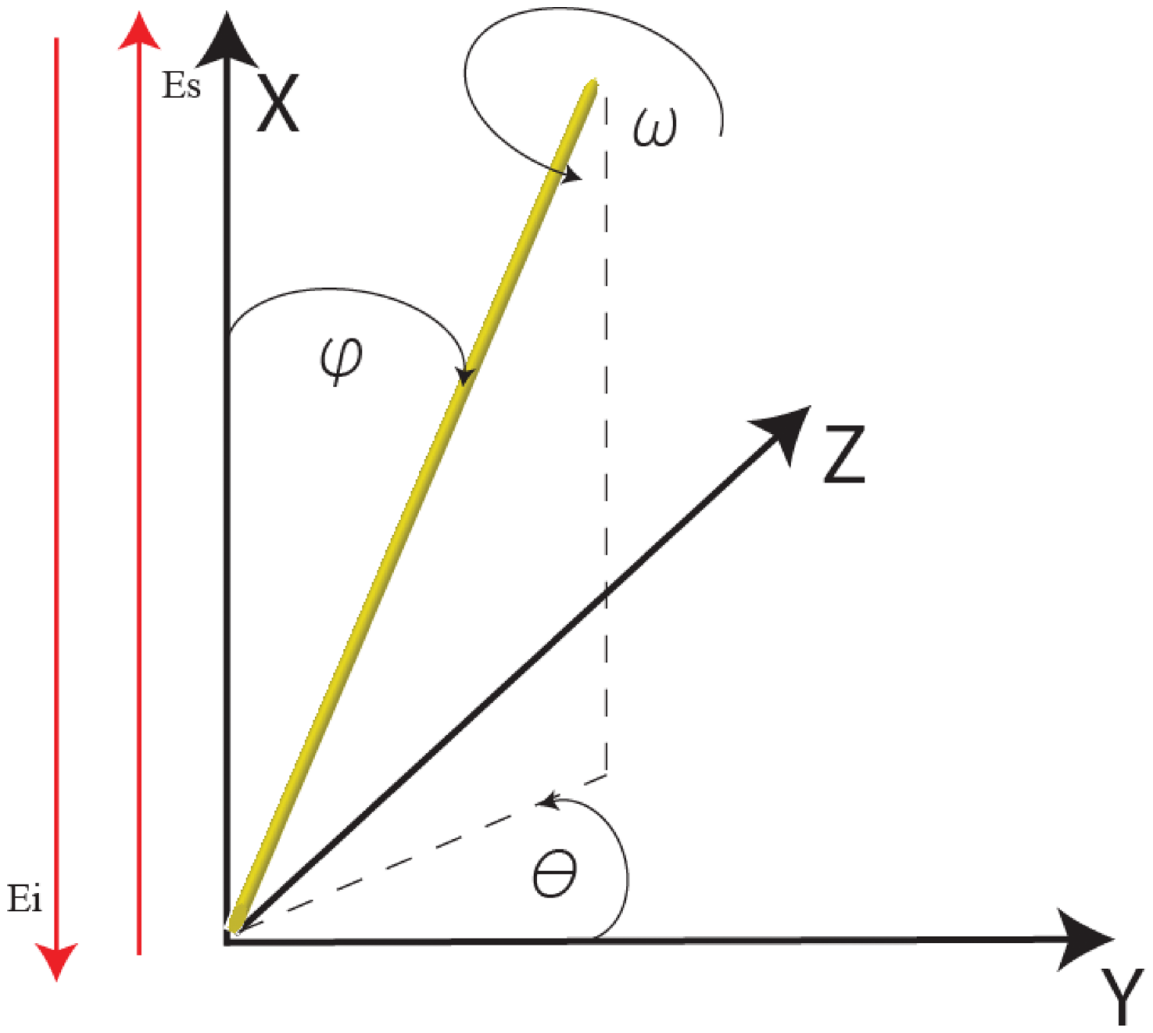
Global coordinate system and the Euler angles that describe the position of the molecular structures of collagen-like peptide and alpha helix. The directions of propagation of the incident and scattered beam (E_i_ and E_s_) are represented by the red arrows parallel to the X axis while yellow bar represents the position of molecular structures. The “in plane” rotations are performed in the plane YZ and the “out of plane” rotations are performed in the ZX plane.

The theoretical anisotropic response of the amide I band at different laser polarization angles was calculated for a single alpha helix and several collagen-like peptide structures [10]. Four different crystal structures of a collagen-like peptide and one alpha helix were selected in the Protein Data Bank (PDB, ) with the following identification numbers: for collagen-like peptides (1CAG [28], 1BKV [29], 1CGD [30], 1QSU [31]) and for an alpha-helix section of the protein: (1 XQ8 [32]). Matlab© and Mathematica© scripts were developed to visualize and rotate the peptide molecule around the origin of the global coordinate system adopted in the evaluation of the atom positions in the literature [29], calculating the new atomic coordinate at every new position. The global coordinate system and the Euler angles that describe the position of the molecular structures are explained in Fig. 2 and the axis of the alpha helix structure was calculated by the program HELFIT [33].

The evaluation was done at different positions of the structures in the global coordinate system that correspond to “in plane” and “out of plane” rotations. The “in plane” rotations are performed in the plane YZ i.e. that of the sample surface, and the “out of plane” rotations are performed in the XZ plane. The direction of propagation of the incident and scattered energies (E_i_ and E_s_) is along the X axis.

As in Tsuboi and Thomas [34], we use the amide I Raman tensor, T_local_ measured for aspartame. This tensor is oriented such that the x-axis lies in the peptide group plane and at 34° with respect to the C = O bond. The local tensor has the following ratios of its diagonalized : α_xx_/α_zz_ = 20 and α_yy_/α_zz_ = 4. The atomic coordinates of the amide groups were extracted from the crystal structures, and used to write the Raman tensor, T_global_, of each amide group in the global coordinate system using:
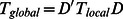
(1)where D’ is the matrix of direction cosines that transform the local coordinate system to the global, and the prime indicates the transpose. The relative Raman amide I response for the entire molecule was calculated using the following equation:
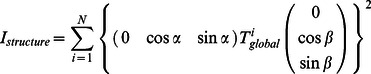
(2)where N corresponds to the number of peptide units present in the collagen crystal structure, α and β are the angle of the analyser and polarizer respectively in the YZ plane. The α = 0°, β = 0° correspond to the Y axis direction while α = 90°, β = 90° to the Z axis direction. In the experiments no analyser was used, meaning the intensity calculated in equation (2) is integrated over alpha. The results are shown as plots of
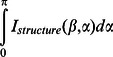
(3)vs the polarization angle β of the incident light.

## Results

**Figure 3:**
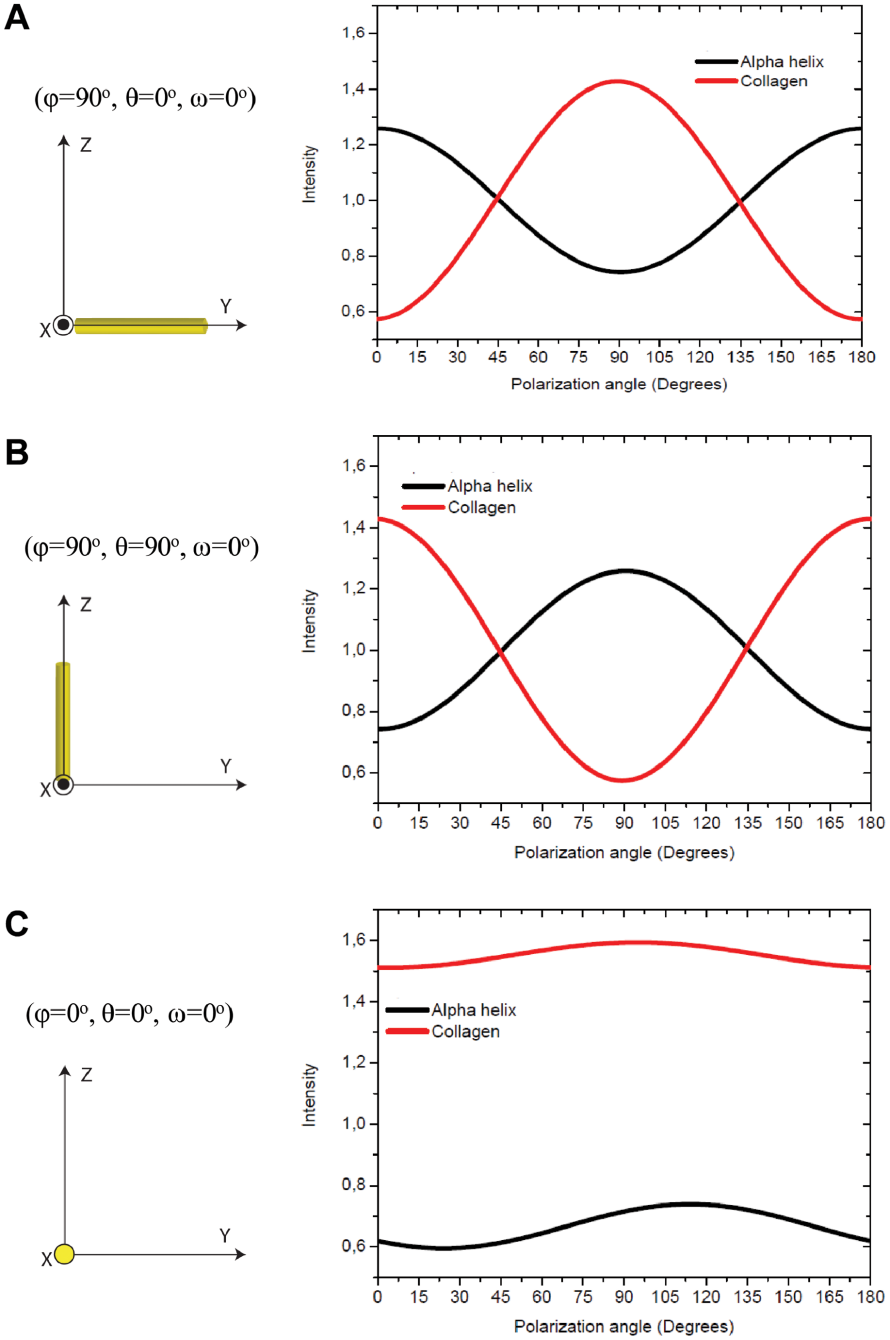
Theoretical prediction of the anisotropic response of amide I band for collagen-like and alpha helix molecules. Normalized anisotropic response of the amide I band of a collagen-like peptide molecule (ID:1CAG) and alpha helix (ID:1XQ8) located at A) (φ = 90°, θ = 0°,ω = 0°) on the plane ZY, B) (φ = 90°, θ = 90°, ω = 0°) on the plane ZY and C) (φ = 0°, θ = 0°,ω = 0°) on the plane ZX. For the collagen-like peptide structure located “in plane” (A and B) the maximum response of the amide I band is obtained when the polarization of the light is parallel to the molecule position, the opposite response is observed for the alpha helix. In the “out of plane” (C) response both structures give rise to a much more isotropic response of the amide I band.

The calculated anisotropic responses of the amide I band in a collagen-like peptide (ID: 1CAG) and alpha helix structure (ID: 1XQ8) are shown in Fig. 3. These are displayed as plots of the integrated intensity over all scattered angles *vs* the polarization angle β of the incident light at three different positions in space: two located “in plane” (φ = 90°, θ = 0°, ω = 0°), (φ = 90°, θ = 90°ω = 0°) and one “out of plane”, perpendicular to the plane YZ in the position (φ = 0°, θ = 0°, ω = 0°).

**Figure 4:**
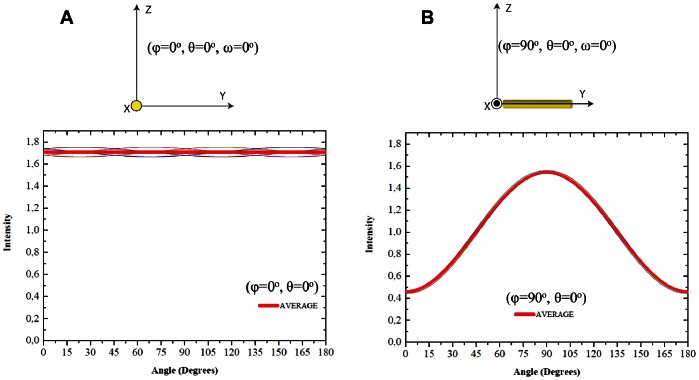
Averaged theoretical amide I response of collagen-like peptide for rotation around the main axis of the molecule. A) Amide I band response of a collagen-like peptide (ID: 1BKV) oriented (φ = 0°, θ = 0°,) plane (i.e. perpendicular to the ZY plane) rotated at different ω angles around the c-axis and its average response. B) Amide I band response of a collagen-like peptide (ID:1BKV) lying in the ZY plane (φ = 90°, θ = 0°) that has been rotated at different ω angles around the c-axis of the molecule and its average response. All graphs are plotted as functions of the polarization angle β of the incident laser beam (according to eq.3).

The relative amide I response is normalized by the mean intensity value of the amide I band that has been placed on the plane ZY. For collagen-like peptides, the minimum intensity response is obtained when the polarization angle of the incident light is parallel to the central axis of the molecule and is maximal when perpendicular. On the other hand, the alpha helix structure exhibits the opposite behaviour: the minimal amide I intensity response is observed when the polarization direction of the incident light is perpendicular to the structure and maximal when parallel. Both structures show a decrease in the anisotropic response of the amide I band as the molecules point more and more “out of plane”. In the collagen-like peptide the mean intensity increases to higher values compared to the maximal response of the amide I band “in plane” rotation, while in the alpha helix structure, the mean intensity decreases even below the minimal response when the structure is placed “in plane”. Some small variation is still seen in Fig. 3C as a function of polarization angle, the reason for which stems just from statistics. For the theoretical analysis, the selected collagen-like peptide (from the protein database) is only a portion of the size of a natural collagen molecule (∼30 amino acid residues in each polypeptide chain of the collagen-like peptide *vs* 1000 amino acid residues in natural collagen). To account for this the amide I response was averaged over 8 angles ω for “in plane” and “out of plane” positions of the collagen like-peptide structure ID:1BKV (Fig. 4). This leads to expected completely isotropic response of the collagen like-peptide molecule for “out of plane” transformation (red curve in Fig. 4A), and very little change in the anisotropic response for “in plane” rotation (red curve in Fig. 4B).

**Figure 5:**
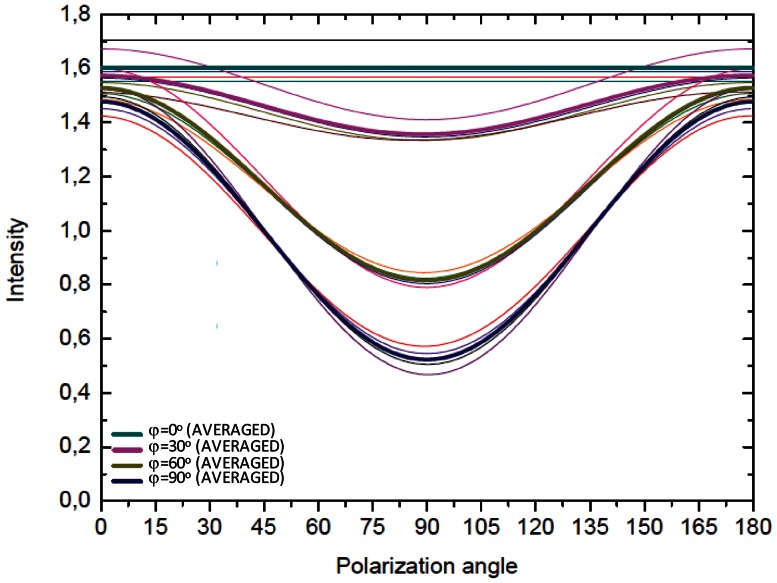
Averaged theoretical amide I response of collagen-like peptide molecules for “out of plane” rotation. Normalized amide I response for four different collagen-like peptide structures (ID: 1BKV, 1CGD, 1QSU) that are rotated in the plane XZ [from (φ = 90°, θ = 90°) to (φ = 0°, θ = 90°)] *vs* the polarization angle of the incident light. The responses have been averaged at angles ω = 0°, ω = 90°, ω = 180°, ω = 270°. All the molecules exhibit a similar trend independent from which collagen-like peptide crystal structure. The average responses for all the selected structures are marked in bold.

Figure 5 compares the normalized amide I intensity (Eq. 3) for four different collagen-like peptide structures (ID: 1CAG, 1BKV, 1CGD, 1QSU) that are rotated in the plane XZ (from (φ = 90°, θ = 90°) to (φ = 0°,θ = 90°)). All the selected collagen-like peptide structures show similar behavior with a reduction in the anisotropic response of the amide I band by decreasing φ. To get an estimate for the general response of collagen, we averaged the data for these different sequences and plotted the results as thick lines in Fig. 5 for several values of the out-of-plane angle φ. For φ = 0 (that is, collagen parallel to the laser beam), the curve is constant not showing any dependence on laser polarization. The more the collagen is oriented away from the laser propagation direction a minimum at β = 90° gets more and more pronounced. It is worth noting that the mean value that would be obtained from averaging over all polarization angles decreases with increasing φ.

**Figure 6:**
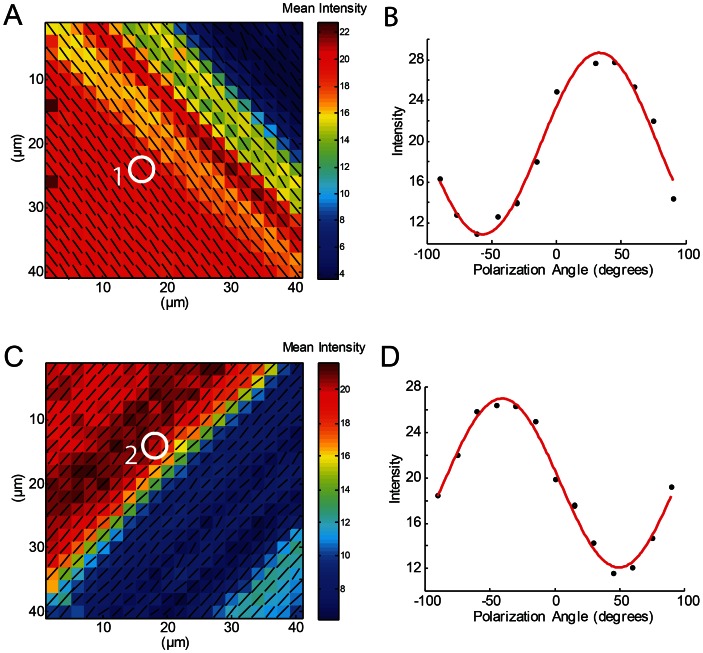
*In situ* PRS mapping of the collagen orientation in −45 and 45 degrees tilted dried RTT. (A) and (C) show maps obtained by fitting thirteen Raman images collected with different polarization angles of the incident laser light. The direction of arrows indicates the orientation of collagen molecules, their length represents the amplitude of the fitting curve, and the color code is the average intensity of the amide I band. (B) and (D) are example of experimental points extracted from the area marked in (1) and (2), respectively.

In order to verify the theoretical results of the spatial response of the amide I band in collagen based structures, PRS experiments on dry rat tail tendon model were performed (see Fig. 1). The system is characterized by highly oriented collagen fibrils. Extracted tendon fascicles were pre-stretched to remove characteristic crimp structures and dried under tension to obtain perfectly aligned collagen molecules. Figure 6A shows collagen orientation map obtained by fitting thirteen amide I intensity images collected with different polarization angles of the incident laser light [25]. The RTT was placed at θ = −45°, φ = 90° and θ = 45°, φ = 90° to measure the anisotropic response for “in plane” fibers using the mapping methodology reported in the literature [25]. The vectors in the map are uniformly oriented around θ = −45° (Fig. 6A) and θ = 45° (Fig. 6C), the only parameter changing in the fitting results is related to the variation in mean intensity of the signal associated to the different density of collagen in various regions of the sample. Intensity values in function of orientation of incident laser polarization for the Area 1 and 2 are plotted in Fig. 6B and D, respectively.

**Figure 7:**
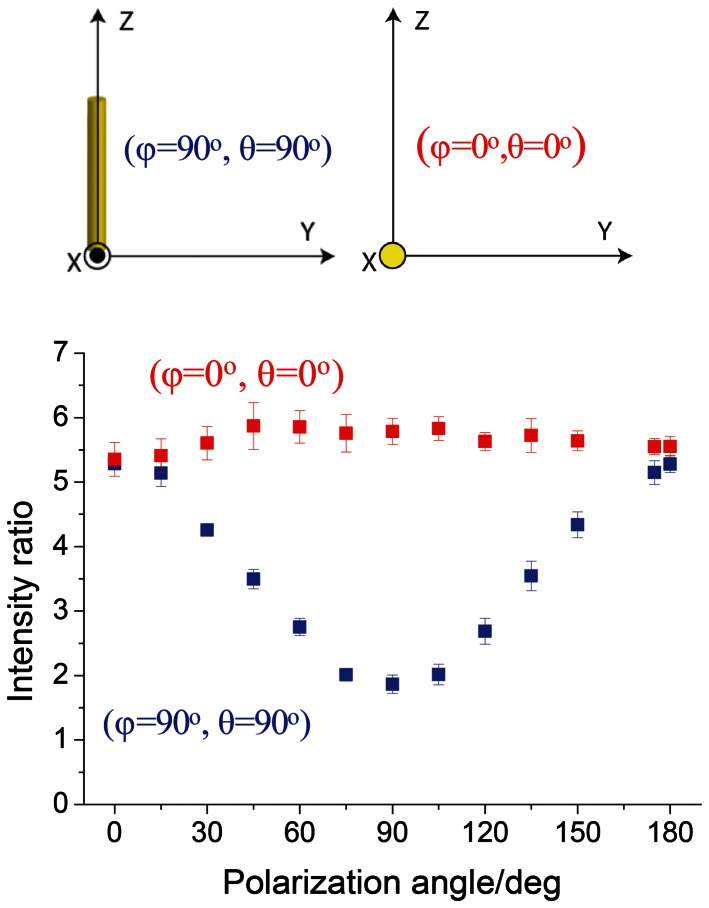
Experimental PRS results for RTT fibers in both “in plane” and “out of plane” configuration. Sinusoidal anisotropic response was found for fiber placed at φ = 90°, θ = 90° (collagen parallel to Z-axis, “in plane” configuration) with maximal intensity when the polarization of the light is perpendicular to the fiber and minimal when parallel. In the case of φ = 0°, θ = 0° configuration (collagen parallel to laser beam, “out of plane” configuration) isotropic response was observed.

Single point PRS experiments on RTT were performed with further two geometries; “in plane” configuration where sample was placed along the z axis ((φ = 90°, θ = 0°), and “out of plane” with sample placed along the x-axis (φ = 0°, θ = 0°). RTT fibers were dried and mounted such that the long axis was oriented parallel to the incident beam. The intensity ratio of amide I band by respect to orientation independent C-C stretching band was measured and plotted in function of the orientation of incident laser polarization (Fig. 7). The clear difference of anisotropic response was observed for two geometries.

## Discussion

In the present work, we have performed a theoretical study based on the classical Raman theory to understand the amide I band response of collagen-like peptide molecules at different spatial orientations. Furthermore, the collagen triple helix was compared with the amide I response of alpha helix using the same methodology. The calculation of the theoretical responses of the amide I band in collagen-like peptide molecules and alpha helix allowed us to explain several features observed in the standard collagen-based materials like RTT. This work was done under the assumption made by Tsuboi et al. [34] that the tensor that describes the amide I band in a single aspartame crystal can be transferred to other biological structures that contain many peptide groups. The length of the peptide-molecule-peptide is an important aspect for sample crystallization; a longer collagen fragment is more flexible, but also more difficult to crystalize [35].

In comparison, each polypeptide chain of natural collagen consists approximately of 300 to 330 units of Gly-X-Y (where X usually corresponds to proline and Y to 4-hydroxyproline) or 1000 amino-acid residues. In the five crystal structures selected, each single polypeptide chain of collagen like-peptide contains around 10 triplets (30 amino-acid residues). The complete response of the amide I band in collagen is given by the summation of all amide I scattering centres present in the collagen structures and due to the short length of the collagen-like peptide structure the obtained amide I response will be only partial. Thus to mimic the response of many parallel collagen structures, the amide I response of the full length collagen molecule was estimated by the average of the response of the five studied short collagen-like peptides upon their rotation around their c-axis. This is important, as although the molecules within the confocal volume are aligned along the fibre direction they are likely to be disordered with respect to their angular orientation about their c-axes. The average response was calculated for two positions of one collagen-like peptide structure: one out of plane (φ = 0°), where the average of the amide I response of the rotating molecule around the axis gives a straight line that reflects the completely isotropic response; the other lying on one of the axes of the “in plane” rotation. The “in plane” amide I band response at different polarization angles of the incident light shows a sinusoidal behaviour. Intensity is minimal when the collagen molecule is parallel to the polarization angle, and maximal when perpendicular. For the “out of plane” rotation (φ = 0°) the amide I response becomes totally isotropic.

A similar evaluation was done for the amide I band response at different spatial orientations of the selected alpha helix structure. Interestingly, the amide I intensity at different polarization angles of the alpha helix structure rotated “in plane”, which was 18 amino acid residues long, was found to have minimum intensity when the polarization angle of the incident light is perpendicular to the alpha helix, and maximum when parallel. This behaviour is opposite to the amide I band response of the collagen-like peptides. The reason for that is mainly due to the different orientations in the carbonyl group (C = O) of the peptide groups between collagen-like peptides and alpha helixes; in alpha helices these groups are located mainly parallel to the axis of the structure (organization required for the formation of hydrogen bonds responsible for the stability of helical structure), while in collagen-like peptides the carbonyl groups are located preferentially perpendicular to the axis [25]. Latter aspect explains also the opposite response of the amide I band for the “out of plane” rotation (Fig. 3C). Even though both structures give rise to a more isotropic response, alpha-helix structure displays a decreased average intensity whereas for collagen an increase of average intensity was observed.

These results suggest that the amide I tensor for alpha helix is a prolate spheroid while for collagen is oblate. At this point, it is worth noting that it is assumed that the amide I tensor from single peptide unit obtained from aspartame is not modified by the formation of the hydrogen bonding inter-chain between the N-H⋅⋅⋅⋅O = C when is transferred to collagen and alpha helix structures as demonstrated in polarized Raman studies of both polypeptide and protein alpha helix [36], [37].

In context of mapping collagen orientation in tissues, dried RTT vas placed at −45 and 45 degrees and orientation of collagen molecules was obtained by fitting procedure reported in literature. The calculated collagen orientation maps (Fig. 6) reflect clearly the −45° and 45° fiber direction in the investigated area.

The anisotropic response of the amide I band for collagen fibrils located “out of plane” with respect to the plane of observation can be easily followed in RTT (Fig. 7). The anisotropic response decreases with the mean intensity increasing, result that is in excellent agreement with the theoretical predictions (Fig. 5).

It is clear that the measurements of the mean intensity of the amide I band using linearly polarized incident laser light can lead to errors in composition and analysis of the collagen based tissues as high as 50–60%. However, the anisotropic response of the amide I band in well-organized collagen-based materials can be used as a methodology for characterization of collagen fibres 3D spatial orientation based on the fitting of the amide I band response at different polarization angles of the incident light. The methodology presented here, supported by theoretical predictions, opens new possibilities in imaging and evaluation of structure-function relations in collagen based biological tissues.

In this work, a theoretical approach was used to predict the PRS anisotropic response of the amide I band of collagen and alpha helical structures by respect to the polarization angle of the incident laser light. The sinusoidal behaviour of the anisotropy is directly related to the global Raman tensor for the protein structures. Using this approach we calculated the Raman intensity response for different orientation configurations of the collagen and alpha helical molecules in space (“in plane” and “out of plane” rotations). The results paved the road for a 3D mapping of protein structures characterized by cylindrical symmetry. The proof of principle was confirmed by measuring polarized Raman signals of highly organized collagen based tissue, namely RTT. Our results show remarkable agreement of experimental data with theoretical predictions and the methodology proposed here has proven to be very useful in the evaluation of 3D orientation of collagen molecules in the space in organised tissues.
